# Characterization of Phylogroups, Virulence Factors and Antimicrobial Resistance in Canine Pyometra‐associated *Escherichia coli*

**DOI:** 10.1002/vms3.71171

**Published:** 2026-08-11

**Authors:** İnci Başak Müştak, Murat Onur Yazlık, Beyza Nur Şitvan, Hamit Kaan Müştak, Ufuk Kaya

**Affiliations:** ^1^ Faculty of Veterinary Medicine, Department of Microbiology Ankara University Ankara Turkey; ^2^ Faculty of Veterinary Medicine, Department of Obstetrics and Gynecology Ankara University Ankara Turkey; ^3^ Faculty of Veterinary Medicine, Department of Biostatistics Hatay Mustafa Kemal University Hatay Turkey

**Keywords:** antibiotic resistance, dog, *Escherichia coli*, phylogenetic group, pyometra, virulence factors

## Abstract

The presence of multidrug‐resistant extraintestinal pathogenic *Escherichia coli* (ExPEC) in pets poses a significant threat to animal health and represents a potential source of antimicrobial resistance with zoonotic transmission to humans. A total of 56 pyometra swab samples from dogs were analysed for the presence of *E. coli* using standard microbiological procedures. Phylogrouping of the *E. coli* isolates was performed using the quadruplex, and 12 virulence genes were detected by single polymerase chain reaction (PCR) method. Antimicrobial susceptibility testing was conducted against 12 antimicrobial agents in five different classes using the Kirby–Bauer disc diffusion method. *E. coli* was isolated from all 56 swab samples of pyometra cases using 5% ovine blood agar and MacConkey agar, and identified with Eosin Methylene Blue agar, Methyl Red and Voges–Proskauer tests. *fimH* was found in all strains. The most common virulence genes were *fyuA* (96.4%), *usp* (83.9%), *sfa* (73.2%), *hlyA* (69.6%), *cnf1* (69.6%), *pap*GIII (64.2%), and *papC* (58.9%). The least common virulence gene was *iucD*,while none of the strains carried *afa* and *pap*G allelic variants (*pap*GI and II). A total of 83.9% of isolates belonged to phylogenetic group B2. All isolates were resistant to amoxicillin/clavulanic acid, ampicillin and lincomycin but sensitive to amikacin. The multidrug resistance (MDR) rate was 58.9% (33/56 isolates). MDR and multiple antibiotic resistance index (MARI) scores were similar between the groups (*p* > 0.05). The phylogenetic analysis revealed that resistant isolates were found across all detected phylogroups, with strains from different phylogroups sharing the same virulence genes. *E. coli* from pyometra cases in companion animals is largely associated with resistance and possesses virulence genes enabling efficient colonization and carriage.

## Graphical abstract

This study characterized 56 *Escherichia coli* isolates obtained from canine pyometra, which predominantly belonged to phylogroup B2 and harboured multiple virulence genes, with high multidrug resistance rates indicating potential clinical and zoonotic risks.

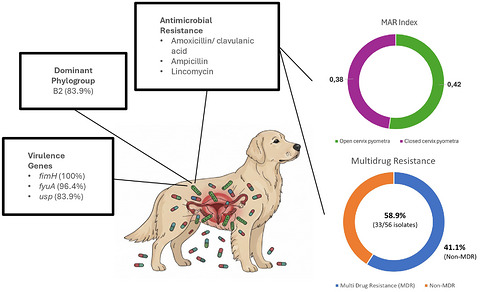

**Abbreviations**
ExPEC: extraintestinal pathogenic *Escherichia coli*
MARI: multiple antibiotic resistance index
MDR: multidrug resistance
PCR: polymerase chain reaction
UPEC: uropathogenic *Escherichia coli*
UTIs: urinary tract infections
UV: ultraviolet
VF: virulence factor

## 1 Introduction

Pyometra is a common disease in female dogs. It is characterized by endometrial hyperplasia, uterine inflammation and accumulation of purulent exudate (Hagman 2018; Woźna‐Wysocka et al. 2021; Xavier et al. 2023). If left untreated, pyometra can have consequences ranging from endotoxemia, sepsis and septic shock to death (Hagman 2022). The disease occurs in the luteal phase of the oestrous cycle, when increased progesterone levels play an important role in infections by encouraging opportunistic pathogens (Hagman 2018). Under the influence of progesterone, the uterine local cellular immune system becomes suppressed, and together with increased age, subacute/chronic degenerative changes in uterine tissue enable bacteria to adhere, colonize, grow and release toxins (Chen et al. 2003; Ishiguro et al. 2007; Leitner et al. 2003; Pretzer 2008). The aetiology of pyometra is complex with hormonal and bacterial origins (De Cock et al. 1997; Dhaliwal et al. 1999; Hagman 2018; Xavier et al. 2024). Of the many bacteria associated with pyometra, *Escherichia coli*is the most frequently isolated (Lopes et al. 2020; Rocha et al. 2022). The prevalence of canine pyometra changes according to the location. In United Kingdon, between 2006 and 2011, the prevalence of canine pyometra was 2.2% (Gibson et al. 2013). On the other hand, in India, this prevalence is 7.5% (Pitroda et al. 2025) and in Sweeden 20% (Jitpean et al. 2012).

In most animals, *E. coli* is part of the normal intestinal flora. *E. coli* is genetically divided into three main groups: commensal, pathogenic (enteric or diarrheagenic) and extraintestinal pathogenic (ExPEC) (Da Silva and Mendonca 2012), of which ExPEC *E. coli* is the most common pathogen isolated in canine pyometra (Sroithongkham et al. 2024; Xavier et al. 2022). *E. coli* strains isolated from canine pyometra show similarities with urinary tract infections (UTIs), probably because both infections are caused by intestinal flora or agents ascending from the vaginal canal (Hagman 2022). Although *E. coli* resident in the canine intestine are associated with pyometra, uropathogenic *E*. *coli* (UPEC) strains differ from commensal intestinal bacteria in terms of their specific virulence factors (VFs) (Siqueira et al. 2009).

Phylogenetic analyses have revealed that *E. coli* strains are divided into four main phylogenetic groups (A, B1, B2 and D) (Clermont et al. 2000), which differ in phenotypic characteristics, such as the ability to metabolize different sugars, antibiotic resistance profiles and growth rate–temperature relationship. Virulent extra‐intestinal strains fall mainly in group B2 but also in group D, whereas most commensal strains are in groups A and B1 (Clermont et al. 2000, 2013). These studies have also led to a better understanding of how pathogenic strains acquire virulence genes (Bingen et al. 1998; Clermont et al. 2000).

The four main phylogroups of *E*. *coli* strains (A, B1, B2 and D) have been differentiated by triplex polymerase chain reaction (PCR) targeting *chuA*, *yjaA* and *TspE4.C2* genes. A new quadruplex PCR, adding the *arpA* gene, enabled identification of eight phylogroups (A, B1, B2, C, D, E, F and *E. coli* clade I) (Clermont et al. 2013). This simple, inexpensive and frequently used method provides information about the phenotypic and genotypic characters, ecological roles and disease‐causing abilities of the strains separated into phylogroups. Knowledge of these features can facilitate the prevention, control and treatment of diseases (Clermont et al. 2013). In canine pyometra, for example, *E. coli* strains are commonly associated with phylogroup B2 (Henriques et al. 2014; Liu et al. 2017; Mateus et al. 2013; Xavier et al. 2022).

*E. coli* strains produce a variety of factors to colonize the host, such as siderophores, adhesins, extracellular products and toxins (Flores‐Mireles et al. 2015; Hagman and Kuhn 2002). Previous studies have detected several important virulence genes, including P fimbria encoded by pap (*papC/G* and three allelic variants of *pap*G), toxins (*hlyA*, *cnf1*), adhesins (*fimH*, *sfa*), afimbrial adhesion (*afa*), aerobactin sidherephore (*iucD*), iron uptake (*fyuA*) and uropathogenic‐specific protein (*usp*) (Liu et al. 2017; Mojaz‐Dalfardi et al. 2020; Osugui et al. 2014; Siqueira et al. 2009).

Cross transmission of ExPEC between animals and humans has been widely reported (Guardabassi 2004; Sidjabat et al. 2009; Sroithongkham et al. 2024), while antimicrobial resistance (AMR) is another concern. Today, drug‐resistant *E. coli* strains are considered one of the most significant clinical challenges affecting both human and animal health (De Oliveira et al. 2020). AMR is a global health concern that threatens the effectiveness of antibiotics and complicates the treatment of infectious diseases (Blair et al. 2015). Importantly, resistance genes may also be present in non‐pathogenic bacteria, such as commensals, and can be transferred to pathogenic bacteria, making healthy animals potential carriers of AMR genes (Djordjevic et al. 2024). In the context of canine pyometra, this highlights the importance of monitoring resistance patterns of isolated bacteria in the infection. Despite the high prevalence of pyometra in companion animals, limited data exist from our region regarding the phylogenetic background, VFs and AMR profiles of *E. coli* isolates. Therefore, we aimed to characterize *E. coli* strains isolated from pyometra of dogs in terms of phylogroup, virulence genes and resistance to the antimicrobials used in the clinic, and investigate the relationship between these factors.

## 2 Materials and Methods

### 2.1 Bacterial Isolation

The *E*. *coli* isolates used in this study were obtained from swab samples taken from the contents of open and closed canine pyometra sent to the Department of Microbiology at Ankara University, Faculty of Veterinary Medicine. A total of 56 pyometra cases from pet dogs were included in the study, consisting of 27 open cervix and 29 closed cervix cases.

Animals with pyometra separated into two groups according to the cervical patency status as open and closed cervix pyometra. The surgical treatment was performed for soflurane‐ and propofol‐based general anaesthesia and total ovariohysterectomy by the ventral midline incision were performed as previously described by Yazlık et al. (2022). Following surgery, the removed uterine tissues were transferred to the sampling room. Uterine horns washed with sterile saline and disinfected with iodine solution. The serosa of uterine tissue was incised with a scalpel and aseptic, and sterile cotton swabs were passed through the incision to collect samples from the uterine content, following the protocol of Yazlık et al. (2022).

All samples were inoculated on 5% ovine blood agar and MacConkey agar (Oxoid) and incubated at 37°C for 24 h. Suspected *E. coli* colonies were further subcultured on Eosin Methylene Blue Agar (Oxoid) at 37°C for 24 h and the colonies were then subjected to morphological examination and biochemical tests including Methyl Red‐Voges Proskauer Medium (MR‐VP Oxoid) for identification *E. coli*. Colonies showing red coloration in MR (pH ≤ 4.4) and yellow coloration in VP tests were considered MR‐positive and VP‐negative, which are typical characteristics of *E. coli*. The strains then were stored in trypticase soy broth (TSB, Merck) with 30% glycerol at −20°C until use.

### 2.2 Detection of Virulence Genes and Phylogenetic Grouping for *E. coli*

Nucleic acid extraction was conducted using the boiling method (Millemann et al. 2000), followed by phylogrouping of the *E. coli* strains using the quadruplex PCR method (Clermont et al. 2013).

PCR analysis was conducted to detect 12 virulence genes: *papC/G*; three allelic variants of *pap*G and *afa*, *sfa*, *hlyA*, *iucD*, *cnf1*, *fimH*, *usp* and *fyuA*. The PCR reaction performed in a total volume of 25 µL containing 0.2 µM of each primer (Table 1), 200 µM dNTP, 2.5µl PCR reaction buffer, 3mM MgCl_2_, 2U Taq DNA Polymerase (Thermo Scientific; EP0402) and 2µL of template DNA. The PCR was performed with an initial denaturation at 94°C for 3 min, followed by 30 cycles of 1 min at 94°C, 60 s at 55°C (35°C for *cnf1* gene), 1 min at 72°C, and a final extension for 7 min at 72°C. The PCR products were analysed by electrophoresis on 1.5% agarose gel (Promega Corporation, USA) and visualized under a ultraviolet (UV) transilluminator.

**TABLE 1:** Primers used for identification of virulence genes.

Target	Primer sequences (5′–3′)	Amplicon size (bp)	Reference
*papC*	F: GAC GGC TGT ACT GCA GGG TGT GGC G R: ATA TCC TTT CTG CAG GGA TGC AAT A	328	(Siqueira et al. 2009)
*pap*GI	F: CAA CCT GCT CTC AAT CTT TAC TG R: CAT GGC TGG TTG TTC CTA AAC AT	692	(Siqueira et al. 2009)
*pap*GII	F: GGA ATG TGG TGA TTA CTC AAA GG R: TCC AGA GAC TGT TCA AGA AGG AC	562	(Siqueira et al. 2009)
*pap*GIII	F: CAT GGC TGG TTG TTC CTA AAC AT R: TCC AGA GAC TGT GCA GAA GGA	421	(Siqueira et al. 2009)
*afa*	F: GGC AGA GGG CCG GCA ACA GGC R: CCC GTA ACG CGC CAG CAT CTC	559	(Johnson and Stell 2000)
*sfa*	F: CGG AGG AGT AAT TAC AAA CCT GGC A R: CTC CGG AGA ACT GGG TGC ATC TTA C	410	(Siqueira et al. 2009)
*hlyA*	F: AAC AAG GAT AAG CAC TGT TCT GGC T R: ACC ATA TAA GCG GTC ATT CCC GTC A	1117	(Siqueira et al. 2009)
*iucD*	F: TAC CGG ATT GTC ATA TGC AGA CCG T R: AAT ATC TTC CTC CAG TCC GGA GAA G	602	(Siqueira et al. 2009)
*cnf1*	F: GAA CTT ATT AAG GAT AGT R: CAT TAT TTA TAA CGC TG	533	(Siqueira et al. 2009)
*fimH*	F: TGC AGA ACG GAT AAG CCG TGG R: GCA GTC ACC TGC CCT CCG GTA	508	(Johnson and Stell 2000)
*usp*	F: ATG CTA CTG TTT CCG GGT AGT GTG T R: CAT CAT GTA GTC GGG GCG TAA CAA T	1000	(Siqueira et al. 2009)
*fyuA*	F: TGA TTA ACC CCG CGA CGG GAA R: CGC AGT AGG CAC GAT GTT GTA	880	(Johnson and Stell 2000)

### 2.3 Antimicrobial Susceptibility Test and MAR Index

The antimicrobial susceptibility test was performed with 12 antimicrobial agents: β‐lactams (ampicillin [10 µg], amoxicillin/clavulanic acid [20 + 10 µg], cefotaxime [30 µg] and cefoperazone [30 µg]), sulfonamides (trimethoprim/sulfamethoxazole [5 µg]), quinolone/fluoroquinolones (nalidixic acid [30 µg] and ciprofloxacin [5 µg]), aminoglycosides (gentamicin [10 µg], streptomycin [10 µg], kanamycin [30 µg] and amikacin [30 µg]) and lincosamide (lincomycin [15 µg]). The test was conducted using the Kirby–Bauer disc diffusion method according to the Clinical and Laboratory Standards Institute's guidelines (CLSI 2022). *E. coli* ATCC 25922 was used for quality control.

The antimicrobial agents tested in this study were selected based on their frequent use in the treatment of canine uterine infections in clinics together with sepsis or not following the previously described articles (Fieni et al. 2014; Figueiredo et al. 2017; Yazlık et al. 2022). Isolates showing resistance to three or more antimicrobial classes were assessed as multidrug resistant strains (Magiorakos et al. 2012).

The multiple antibiotic resistance (MAR) index is determined by dividing the number of antibiotics to which a given isolate shows resistance (*a*) by the total number of antibiotics tested against that isolate (*b*). Thus, MAR = *a*/*b*. A MAR index value of 0.2 or higher is generally indicative of isolates originating from environments with intensive antibiotic application and considered high‐risk contamination sources (Ejiofor et al. 2016; Sandhu et al. 2016).

### 2.4 Statistical Analysis

All analyses were conducted using the Stata/SE 15.1 software. Results were considered statistically significant at *p* < 0.05. The associations among phylogroup (B2, B1 and Clade I), VF (positive/negative), antibiotic resistance, multidrug resistance (MDR) index and group (open and closed cervix pyometra) were determined using the chi‐square and Fisher's exact tests. The Student's *t*‐test was used to compare the MAR index between open and closed cervix pyometra groups.

## 3 Results

### 3.1 Antimicrobial Susceptibility Testing and MAR Index of Isolates

The antibiotic susceptibility test results indicated that all isolates were resistant to amoxicillin/clavulanic acid, ampicillin (β‐lactams) and lincomycin (lincosamide), but sensitive to amikacin (aminoglycoside). The highest resistance rate was against cefotaxime (50%), followed by kanamycin (32.14%), nalidixic acid (30.35%), cefoperazone (21.42%), streptomycin (17.85%), trimethoprim/sulfamethoxazole (17.85%), ciprofloxacin (12.5%) and gentamicin (5.35%). All isolates showed resistance against at least one antibacterial agent (Table 2). As a result, 33 of the 56 isolates (58.9%) were multidrug resistant (Table S1).

**TABLE 2:** Resistance rates of *E. coli* isolates from dogs with open and closed cervix pyometra.

Antimicrobial drug	Open cervix pyometra (no. of isolates)	Closed cervix pyometra (no. of isolates)	Total
Ampicillin	27 (100%)	29 (100%)	56 (100%)
Amoxicillin/clavulanic acid	27 (100%)	29 (100%)	56 (100%)
Amikacin	0 (0%)	0 (0%)	0 (0%)
Cefotaxime	15 (55.5%)	13 (44.8%)	28 (50%)
Cefoperazone	6 (22.2.%)	6 (20.68%)	12 (21.42%)
Ciprofloxacin	3 (11.1%)	4 (13.7%)	7 (12.5%)
Gentamicin	3 (11.1%)	0 (0%)	3 (5.35%)
Kanamycin	9 (33.3%)	9 (31.0%)	18 (32.1%)
Lincomycin	27 (100%)	29 (100%)	56 (100%)
Nalidixic acid	10 (37.0%)	7 (24.1%)	17 (30.3%)
Streptomycin	8 (29.6%)	2 (6.89%)	10 (17.8%)
Trimethoprim/sulfamethoxazole	3 (11.1%)	7 (24.1%)	10 (17.8%)

**FIGURE 1:**
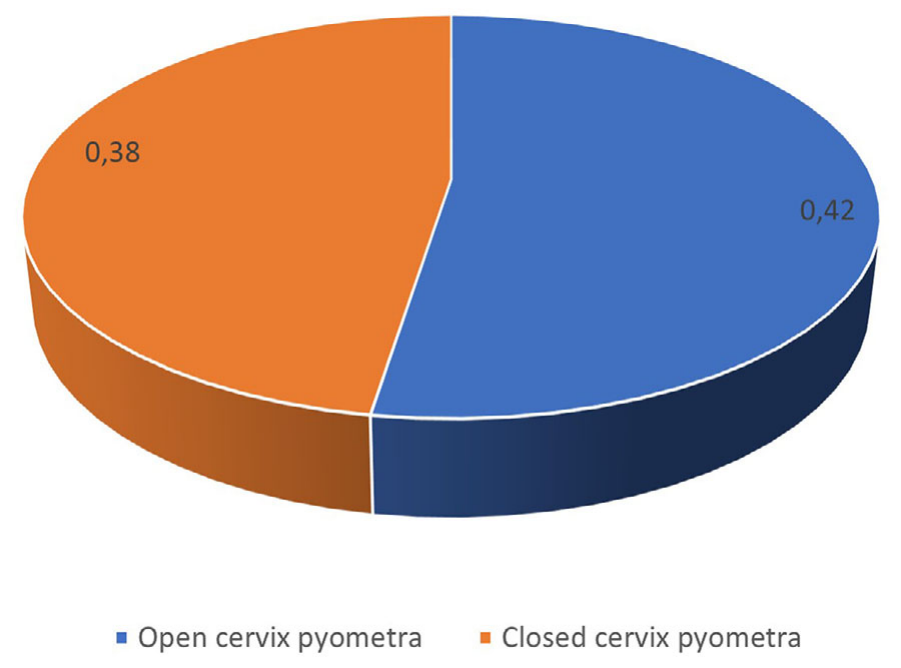
Mean distribution of MAR index of isolates between open and closed cervix pyometra cases.

Despite the different clinical forms, high levels of resistance were observed in both groups. Figure 1 shows the mean MAR index distribution, in open and closed cervix pyometra.

### 3.2 Phylogenic Groups of the Isolates

**FIGURE 2:**
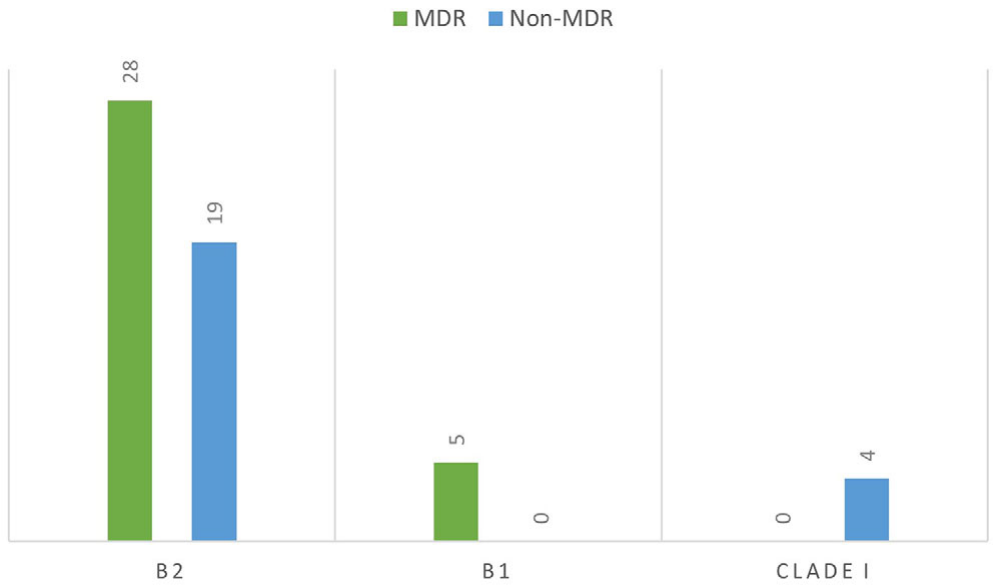
Distribution of *E. coli* phylogroups in canine pyometra isolates by MDR status.

The PCR assay revealed that the 56 *E. coli* isolates from canine pyometra fell into three phylogenetic groups: 47 (83.9%) to B2, 5 (8.9%) to B1 and 4 (7.1%) to Clade‐1. None of the other phylogroups (A, C, D and E) were identified (Figure 2).

Regarding the relationship between the phylogenetic analysis and the isolates’ antibiotic resistance, antibiotic resistant isolates were distributed across all three detected phylogroups (Table S1). Regarding MDR, 84.8% (28/33) of B2 phylogroup strains, 15.1% (5/33) B1 and 0% (0/33) Clade‐1 were MDR. Note that 40.4% (19/47) of B2 phylogroup strains did not have an MDR profile (Figure 2).

### 3.3 Virulence Genotyping

Genotypic virulence characterization of the isolates showed that all strains had at least one gene encoding a VF. Virulence genes associated with adhesion (*fimH*, 100%; *sfa*, 73.2%; *papC*, 58.9% and *pap*GIII 64.2%) were detected in most isolates, whereas afimbrial adhesion (*afa*) and *pap*G allelic variants (*pap*GI and II) were not. The most common virulence genes were *fyuA* (96.4% of isolates) and *usp* (83.9%), which are responsible for iron uptake and uropathogenic‐specific protein, respectively. Thirty‐nine isolates (69.6%) harboured both toxins (*hlyA* and *cnf1)*. The least common virulence gene was *iucD*, which is responsible for aerobactin siderophore VF. This gene was detected in only three isolates.

Seven different combinations of virulence genes were detected. The most prevalent (58.9%) combination pattern was *papC‐pap*GIII*‐sfa‐hlyA‐cnf1‐fimH‐usp‐fyuA* (Table S1). Isolates with this pattern fell into the B2, B1 and Clade‐1 phylogroups.

### 3.4 Descriptive Statistics

There were no statistically significant differences among groups in terms of phylogroups. The presence of virulence genes was similar between open and closed cervix pyometra (*p* > 0.05). MDR and multiple antibiotic resistance index (MARI) scores were also similar between the groups (*p* > 0.05) Except for streptomycin, antibiotic resistance rates were similar between the groups. However, a statistically significant association was found between the *papC* gene and MDR in closed cervix pyometra samples (*p* < 0.05).

## 4 Discussion

In the present study, *E. coli* was isolated from all canine pyometra samples, highlighting its predominant role in this infection. The results were consistent with previous studies showing that E. *coli* is one of the important bacteria involved in pyometra pathogenesis (Castillo et al. 2018; Chen et al. 2003; Lopes et al. 2021; Rainey et al. 2018; Xavier et al. 2022). The isolation rate in our study is higher than that reported in companion animals. For example, Lopes et al. (2021) reported a 57.7% isolation rate among 30 companion animals with pyometra, which underscores the significant role of *E. coli* in the study region. This finding also aligns with reports from different animal species and settings: Castillo et al. (2018) reported *E. coli* isolation in a pyometra case in sheep, Chen et al. (2003) identified *E. coli* in 23 canine pyometra cases, and Xavier et al. (2022) reported *E. coli* in 40 of 72 pyometral dogs. Even in exotic species, such as the Bengal tiger, Rainey et al. (2018) detected *E. coli* in a pyometral case.

The 56 *E. coli* strains isolated from pets with confirmed pyometra were characterized for presence of specific VFs, phylogenetic group, and sensitivity to *E. coli* antimicrobials. The most prevalent virulence genes were *fimH* (100%) and *fyuA* (96.4%). Many other studies have reported that *fyuA* gene is a common gene in both intestinal or extraintestinal *E. coli* isolates (Habibi et al. 2017; Mojaz‐Dalfardi et al. 2020; Yun et al. 2014). In our study, *usp* gene prevalence was 83.9%, which is higher than that recorded for pyometra cases in other studies, specifically 69.2% (Siqueira et al. 2009), 67% (Lopes et al. 2020), 39.1% (Maluta et al. 2014) and 17.1% (Agostinho et al. 2014). On the other hand, the *usp* gene has also been detected in 80% of *E. coli* isolates from human cystitis (Kurazono et al. 2003, 2000). Kurazono et al. (2003) and Parret and De Mot (2002) argue that *usp* plays a role in *E. coli* infectivity and can act as a colicin.

We detected the S fimbriae encoding gene (*sfa*) in 73.2%, which is a similar prevalence to that reported by Chen et al. (2003) and Mateus et al. (2013), but higher than that reported by Siqueira et al. (2009). Like previous studies (Chen et al. 2003; Maluta et al. 2014; Mateus et al. 2013; Siqueira et al. 2009), we did not detect the *afa* gene. Mulvey (2002) reported that the *afa* gene is involved in nephritis in humans and detected in low incidence in canine isolates. These Dr adhesins know receptors on human erythrocytes, which may explain the lower incidence in canine isolates (Mulvey 2002).

We detected the *cnf1* gene at a higher prevalence than previous studies (Chen et al. 2003; Siqueira et al. 2009). However, all our isolates with the *cnf1* gene were also positive for *hlyA*, as reported in other studies of different infections in both humans (Starčič et al. 2002) and cattle (Khalifeh and Obaidat 2022). As genes coding for toxin secretion, *cnf1* and *hlyA* work together to damage the cell and increase infection severity (Diabate et al. 2015; Jahandeh et al. 2015).

We detected a very low prevalence (5.3%) of *iucD*, the gene responsible for aerobactin siderophore VF. Other studies have reported 8% (Chen et al. 2003), 17.3% (Siqueira et al. 2009) and 17.4% (Maluta et al. 2014) prevalences in pyometra isolates. According to Chen et al. (2003), canine *E. coli* isolates may be using a different iron acquisition system then humans and other animals.

We detected a high percentage of isolates with MDR profiles, which is concerning given that the MDR profile of *E. coli* poses a significant risk for both animal and human health (Costa et al. 2008; Pitout 2012; Sidjabat et al. 2009). All isolates were resistant to amoxicillin/clavulanic acid, ampicillin, and lincomycin. These antimicrobials are widely used in clinical treatment in dogs. Hagman and Greko (2005) reported that *E. coli* isolates obtained from canine pyometra showed low resistance to ampicillin and intermediate resistance to amoxicillin/clavulanic acid, while Costa et al. (2008) reported 15% resistance to ampicillin in *E. coli* isolates from faecal samples in healthy dogs and cats. Ghanbarpour and Akhtardanesh (2012) detected 10.71% and 3.57% resistance to ampicillin and amoxicillin/clavulanic acid, respectively, in *E. coli* isolates from canine pyometra.

The high antibiotic resistance in *E. coli*, detected in our study complicates treatment options for infections in both humans and animals. This is particularly concerning as infections caused by *E. coli* in humans who have contact with pets may not respond to these antibiotics, necessitating the use of alternative antibiotic therapies. Since most of the antibiotics used in companion animals are also employed in human medicine, this resistance poses a significant public health. Several factors may contribute to increased antibacterial resistance, such as the misuse of antimicrobials in veterinary practices and cross‐resistance within the same class of antimicrobials or even between different classes of these medications. Monitoring AMR is essential to guide effective treatment strategies in both veterinary and human medicine. For this purpose, antimicrobial susceptibility testing plays a crucial role in identifying the resistance of pathogens, and informs the choice of effective treatment. Although previous studies have reported a relationship between virulence genes and MDR (Cooke et al. 2010; Liu et al. 2017; Moreno et al. 2005; Osugui et al. 2014; Rijavec et al. 2008), a statistically significant association between the *papC* gene and MDR was observed only in closed cervix pyometra samples in the present study. As noted in previous studies, there may be many reasons for this, including virulence gene diversity, animal species, the geographical region where the study was conducted and the antimicrobials tested (Koczura et al. 2013; Liu et al. 2017).

The most frequent phylogroup of the *E. coli* isolates in the present study was B2, in line with previous studies (Henriques et al. 2016; Lopes et al. 2021; Mateus et al. 2013; Xavier et al. 2024). Previous studies have found that ExPEC isolates, which usually belong to the B2 and D groups, harbour more virulence genes (Kawamura‐Sato et al. 2010; Moreno et al. 2005; Rijavec et al. 2008). However, we did not find such an association among phylogroup and virulence genes. Instead, the strains from phylogenetic groups B2, B1 and Clade‐1 had both low and high virulence potential.

## 5 Conclusion

We investigated the virulence genes, resistance to antimicrobials and the phylogroups of *E. coli* isolates in canine pyometra. The majority of *E. coli* isolates were assigned to phylogroup B2. Multidrug‐resistant isolates were more often found in this phylogroup and in general in our study compared with other studies. In this study, most of the VFs found in *E. coli* strains isolated from dogs were also identified in *E. coli* strains causing human infections. These findings support the notion that dogs could serve as reservoirs for *E. coli* pathogenic to humans, due to the close contact between humans and dogs. In this regard, there is a need for more epidemiological studies investigating transmission rates and zoonotic risks. In addition, the high level of AMR observed in present study highlights the necessity for appropriate use of antibiotics in veterinary medicine. Routine microbiological tests and antimicrobial susceptibility assessment should become standard practice to improve effective treatment and prevent the misuse of antibiotics, which leads to increasing resistance. This study was limited by the number of adequate isolates and the lack of genome analysis, which could provide deeper insights into resistance and virulence gene mobilization. However, future studies focusing on molecular mechanisms of resistance and virulence gene transfer will further enhance our understanding of *E. coli* pathogenicity in pyometra and its potential impact on public health.

## Author Contributions

**İnci Başak Müştak**: conceptualization, investigation, writing – original draft, methodology, data curation, formal analysis. **Murat Onur Yazlık**: investigation, writing – review and editing, resources, conceptualization. **Beyza Nur Şitvan**: investigation, data curation, methodology, formal analysis. **Hamit Kaan Müştak**: formal analysis, supervision, writing –review and editing, conceptualization. **Ufuk Kaya**: data curation, formal analysis.

## Funding

The authors have nothing to report.

## Conflicts of Interest

The authors declare no conflicts of interest.

## Supporting information




**Supporting information**: vms371171‐sup‐0001‐TableS1.xlsx

## Data Availability

The data that supports the findings of this study are available in the Supporting Information of this article.
